# Grand challenges in organoid and organ-on-a-chip technologies

**DOI:** 10.3389/fbioe.2024.1366280

**Published:** 2024-02-22

**Authors:** Aleksander Skardal

**Affiliations:** ^1^ Department of Biomedical Engineering, College of Engineering, The Ohio State University, Columbus, OH, United States; ^2^ Center for Cancer Engineering, The Ohio State University, Columbus, OH, United States; ^3^ Cancer Biology Program, The Ohio State University and Arthur G. James Comprehensive Cancer Center, Columbus, OH, United States

**Keywords:** organoids, organ-on-a-chip, biofabrication, biomaterials, personalized medicine, drug screening

## Introduction

Organoids and organ-on-a-chip devices fall under the umbrella of microphysiological systems (MPS), which, in general, can be defined as miniature cell cultures—usually three dimensional (3D) models—that recapitulate aspects of human physiology (Skardal et al., 2016; Clevers, 2019). Today, our field has made significant progress since the days where 2D cell cultures and animal models were our only options as preclinical and basic science experimental model systems. We are privileged to have access to countless cell lines that we can increase the utility of by implementing them in 3D (Prestwich et al., 2007). We have generated biomaterial approaches to create a variety of methods by which to support human patient–derived primary cell–based 3D organoids and tissue constructs. (Mazzocchi et al., 2017). We have merged microfluidic device technology with 3D cell cultures to generate tissue- and tumor-on-a-chip platforms (Bhise et al., 2014). This is a rapidly evolving field. However, adoption of these models—while growing—is still limited given the reliance of biomedical research on 2D cell cultures and animal models. (Maltman and Przyborski, 2010). In this Specialty Grand Challenge, we consider the benefits, the hurdles, and the current implementation and future directions of MPS.

## Embracing complexity

**FIGURE 1:**
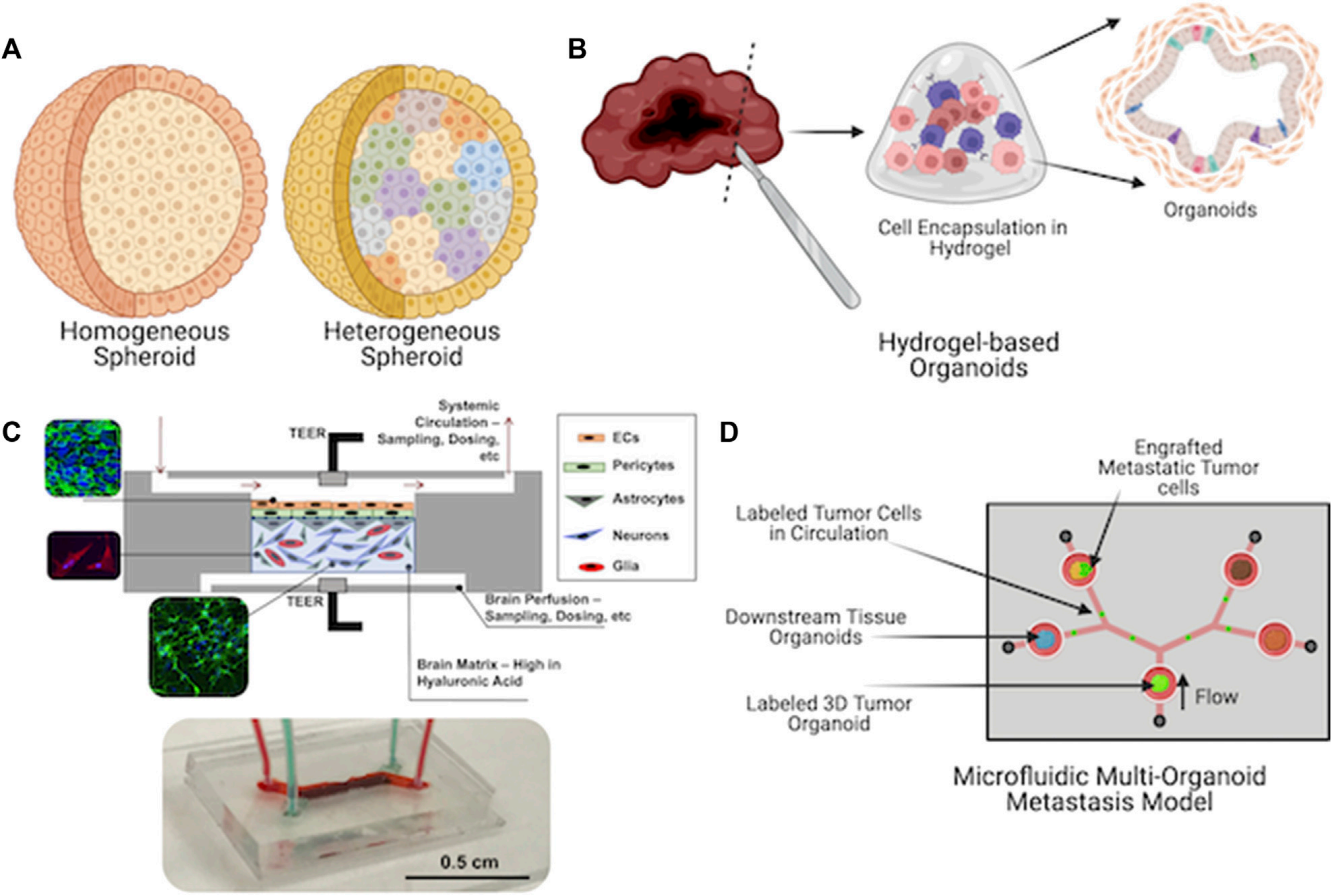
Current microphysiological systems (MPS) span a range of form factors. They range from relatively simple **(A)** spheroids to **(B)** self-organizing organoids to **(C)** engineered microfluidic device-based organ-on-a-chip platforms (a blood-brain barrier system is shown) and finally to **(D)** microfluidic device-based platforms that incorporate multiple tissue types to enable secreted factor or cellular communication between multiple tissue types (a metastasis-on-a-chip platform is shown).

Three dimensional approaches to cell cultures, such as organoids, hydrogel-based constructs, and tissue chips, allow for unprecedented support of cell types that previously were not easy to maintain in 2D cell cultures. As a prime example, only 10–15 years ago, primary human hepatocytes (PHH) were seen as the gold standard for pharmaceutical drug compound toxicity screening. However, the cultures used at the time were either 2D cultures on tissue culture plastic or collagen hydrogel sandwich cultures. Previously, cells would die in about 10 days, during which their functional capabilities (drug metabolism, albumin, and urea production, etc.) would deteriorate drastically within a day or two. In the latter, the collagen sandwich cultures further extended PHH viability, but after 2 or 3 weeks, viability and functionality would quickly deteriorate also. Circa 2012–2015 and onwards, numerous novel biomaterial and 3D culture techniques were invented and deployed, which have made PHH culture relatively straightforward. Examples include integration of decellularized liver extracellular matrix (ECM) into hydrogel biomaterials to provide supporting extracellular matrix (ECM) components and biochemical factors to PHHs (Skardal et al., 2012). Spheroid cultures—both PHH alone and PHHs combined with other liver cells—enabled crucial cell-cell interactions that dramatically improved PHH viability and function (Skardal et al., 2017; Rajan et al., 2020a; Skardal et al., 2020). Today, we have seen numerous liver-on-a-chip devices with impressive *in vivo* liver-like functionality, albeit appropriately volumetrically scaled down (Kang et al., 2015; Knowlton and Tasoglu, 2016; Bauer et al., 2017). These advancements were made by efforts to move past simplistic cell cultures, and to rather recapitulate the complexities of native tissue microenvironments. Today our MPS portfolio comprises a range of form factors, including relatively simple 3D models such as homogeneous cell spheroids, organoids maintained in ECM hydrogels, organ- and tumor-on-a-chip systems, and integrated multi-tissue “body-on-a-chip” platforms (Figure 1).

We have seen similar significant advances in other areas of biomedical research, but probably most impressively in the field of cancer research. The adoption of organoids, alternative 3D cultures, and organ-on-a-chip systems have rapidly accelerated in cancer research, perhaps more than in any other disease-specific research space (Shroyer, 2016; Devarasetty et al., 2018; Gr et al., 2019). *In vitro* cancer MPS occupy spaces in a wide range of cellular, physical, biomaterial, and device-based complexities. Simply moving a common cancer cell line from a 2D tissue culture plastic to a 3D ECM hydrogel matrix can do wonders. A cell line thought to be somewhat artificial can take on highly relevant disease-specific characteristics and serve as a useful research tool (Aleman and Skardal, 2018; Dedhia et al., 2023; Nairon and Skardal, 2023). This is particularly true in rare cancers where patient-derived biospecimen availability for research might be limited.

Utilization of patient-derived cell populations—inherently heterogeneous and more complex than cell lines—can further enhance MPS, in cancer and elsewhere (Vlachogiannis et al., 2018; Ooft et al., 2019). In cancer, the heterogeneity of patient-derived tumor samples can be embraced. The tumor microenvironment (TME) contains many types of cells that are not derived from the tumor itself. These include fibroblasts, immune cells, and many others (Votanopoulos et al., 2019a; Li et al., 2020). As a result, heterogeneous biospecimens from tumors and other tissues are a gift to researchers. They enable the generation of organoids and organ-on-a-chip systems where one can now query biological phenomena such as inflammation and fibrosis (Nairon et al., 2022), and how changes from these phenomena influence disease progression, such as metastasis in the case of cancer (Devarasetty et al., 2017; Devarasetty et al., 2020; Dominijanni et al., 2020). One challenging aspect of *in vitro* tumor models has been integrating immune cells. This has traditionally been difficult, as many of the immune cells would die off, while tumor cells and stromal cells would survive in cell culture conditions of years past. However, more advanced ECM hydrogel systems, new tissue culture media formulations, and increased cellular heterogeneity have provided increased support for various immune cell types. For example, our hyaluronic acid hydrogel platform has successfully supported T cell maintenance and function within melanoma, sarcoma, Merkel cell carcinoma, and appendiceal cancer patient-derived tumor organoids (Votanopoulos et al., 2019a; Votanopoulos et al., 2019b; Forsythe et al., 2022a; Forsythe et al., 2022b). With resident immune cells, immunotherapies such as immune checkpoint blockade therapies can be explored (Votanopoulos et al., 2019a; Forsythe et al., 2022a; Forsythe et al., 2022b). Likewise, cellular immunotherapies have been assessed using such *in vitro* model systems (Dominijanni et al., 2020; Yuki et al., 2020). Both chemotherapies and immunotherapies therapies can be tested in such systems, the results of which can potentially be used in the clinic to inform patient-specific therapeutic decisions, something that we have been fortunate to have performed (Votanopoulos et al., 2019a). Efforts are underway at numerous institutions and medical centers to integrate MPS-based diagnostics with clinical decision making.

Ultimately, we can also use these platforms to combine multiple organoids or 3D cultures into serial or parallel recirculating fluid circuits, enabling crosstalk by secreted factors between 3D constructs, or even cellular transport through circulation to different tissue types (Esch et al., 2014; Esch et al., 2016; Skardal et al., 2017; Aleman and Skardal, 2018; Skardal et al., 2020). These multi-tissue MPS have been relatively limited compared to single tissue type MPS, but are rapidly advancing. To date, there have been several interesting studies demonstrating the potential of these technologies. A clever “body-in-a-cube” system comprised of stacked planar tissue chips supported GI tract, liver, bone marrow, and kidney cell lines, with viability percentages ranging from 80% to 95% (Chen et al., 2020). Ronaldson-Bouchard et al. recently demonstrated the development of a liver, heart, bone, and skin body-on-a-chip connected by vascular flow. Importantly, this system moved away from cell lines, instead using a mix of induced pluripotent stem cell (iPSC)-derived cells and primary cells for the most part. This platform remained viable with appropriate tissue-specific phenotypes for 4 weeks and was used to evaluate pharmacokinetic and pharmacodynamic profiles of doxorubicin (Ronaldson-Bouchard et al., 2022). Our lab was fortunate to be part of a team of researchers that first produced a three-tissue liver, lung, and cardiac platform, in which we showed several examples of crosstalk between tissue types following the introduction of several drug compounds (Skardal et al., 2017). Later, we expanded to a six-tissue “body-on-a-chip” platform housing liver, lung, cardiac, vascular, testis, and brain organoids and tissue constructs. This represented a significant increase in biological complexity, which we initially anticipated to be a significant hurdle for keeping each tissue type viable in the integrated system. Interestingly, despite being comprised of over 15 distinct iPSC-derived or primary cell types, a relatively simple tissue culture media could be used. After some inflammatory biomarkers were observed in the initial days of culture, over time the system self-regulated itself through conditioning of the media, and the viability and functionality remained high for 4 weeks (Skardal et al., 2020). These multi-MPS highlight the advancements in complexity that have been made in the field of organ-on-a-chip technology.

## Limitations holding us back

### Biomaterials

While modern biomaterials have been available for over a decade and a half for tissue engineering applications, the development of new, truly biomimetic biomaterials has lagged behind advances in other areas of science, such as tools for genomics and molecular biology. The term “modern” refers not to biomaterials that are not inert, like metals and ceramics for medical devices of the past, but to biomaterials that mimic the extracellular matrix and dynamically interact with cells (Williams, 2019; Williams, 2022). Despite the fact that advances are being made regularly in the realm of ECM-mimicking hydrogel biomaterials, the fact remains that the vast majority of users fall back on outdated or problematic materials. These include overly simplistic single component collagen or gelatin hydrogels that fail to provide a heterogeneous ECM composition, alginate biomaterials that mammalian cells do not recognize, as well as Matrigel and other basement membrane extract-based biomaterials (Prince et al., 2022). The latter are derived from murine sarcomas and are thus inconsistent and essentially black boxes in terms of composition. As a result, they can introduce countless confounding variables into otherwise well-designed experiments (Li et al., 2010). To be successful, our field requires engineered biomaterial systems that are well-defined, while also recapitulating some aspects of the heterogeneity of native ECM composition.

### Biofabrication technologies

When we use the term “biofabrication” many in our field immediately think 3D bioprinting (Mazzocchi et al., 2019; Sun et al., 2020). 3D bioprinting is certainly a mainstay within biofabrication, which can be used to rapidly bioprint small sized 3D tissue and tumor organoids and constructs. However, in the context of 3D models such as organoids and organ-on-a-chip platforms, there exist a much broader collection of engineering techniques that can be—and indeed are—deployed to generate 3D *in vitro* models. For example, 3D extrusion or inkjet bioprinting is essentially useless if one is working with an already fabricated closed loop microfluidic device-based tissue chip. Fortunately, other creative techniques have been used to introduce 3D cellular constructs within such devices, despite the lack of direct access to the interior volumes of the devices. Specific examples include *in situ* photopatterning of cell-containing hydrogel precursors within the device channels or chambers using photomasks or direct injection through the device walls or roof with very small diameter syringe needles (Skardal et al., 2015; Rajan et al., 2020a; Rajan et al., 2020b). We can expect a wide variety of creative solutions to biofabricate 3D tissue and tumor constructs in easily accessible environments such as well plates, as well as in more limited environments such as organ-on-a-chip-supporting microfluidic devices.

### Regulatory policies

Utilization of organoid and organ-on-a-chip platforms is increasing for a variety of applications. As evidenced by the decision of the United States’ Federal Drug Administration (FDA) to remove the requirement for animal model testing in the drug development pipeline, interest in using bioengineered human cell-based models has become far more widespread than in the past (Teli et al., 2023). However, outside of drug development, MPS can also be used in the context of personalized medicine, as alluded to above. Indeed, we and others have used treatment-response data from tumor organoids to influence patient care, even extending the lifespan of patients (Votanopoulos et al., 2019a). However, despite demonstrating such a powerful use of MPS, due to regulatory hurdles, these occurrences remain rare and are often limited to when a patient has no alternative options. If we could deploy organoid technologies for more patients sooner, one could envision improvements in clinical treatments based on empirical data generated using patient-specific diagnostic treatment screening. However, organoid-based diagnostics are still rare. Such practices need to be approved by regulatory bodies, such as the FDA in the United States or the European Medicines Agency in Europe, and such approval processes are complicated and difficult. Moreover, even if approved, such diagnostic practices require designated facilities that are federally regulated. In the United States, these facilities are Clinical Laboratory Improvement Amendments (CLIA)–regulated laboratories, and not all institutions or medical centers have these facilities. While these regulations exist to keep patients safe, they also serve as barriers that limit the deployment of new technologies that have the potential to make significant positive clinical impacts.

### Organoids versus organ-on-a-chip

In addition, it should be noted that the different MPS form factors that have been discussed here—spheroids, organoids, organ-on-a-chip, and body-on-a-chip—come with additional challenges that vary between them. For example, a cell line–based spheroid is simple to create, is generally inexpensive, and is often a more generic representation of a given human physiology or disease state. It enables easy adoption by researchers. However, since the different MPS form factors are quite homogeneous, and again, somewhat generic, they fail to represent individual humans when they are deployed in applications such as drug development or toxicity testing. Organoids can solve this problem as they often do represent a particular tissue of a particular human patient from whom the cells were isolated. When deployed in the applications above, the outcomes stand a much higher chance of representing individual humans, although it should be noted that genetic drift can occur over time in an organoid culture, as with any other cell culture. However, new challenges arise. A patient-derived organoid only represents that patient, and perhaps other genetically similar persons. As a result, to understand how a new drug compound or toxin impact the population, organoids from a large pool of individuals are needed. At this point, logistical challenges can arise. Access to biospecimens from many individuals are needed, which can be significantly difficult, requiring institutional approval to perform human subject research, as well as the facilities necessary to process human tissue. Lastly, these primary cell-based organoids and 3D tissue constructs are generally more difficult to maintain *in vitro* for long periods of time and often require tissue culture media recipes specific to tissue or even cell type or biomaterial support systems.

Organ-on-a-chip and multi-tissue organ-on-a-chip systems, or “body-on-a-chip” systems, rely on microfluidic devices, which creates additional specific challenges to manage. Beyond the cell-based challenges described above, which certainly still apply, the device hardware adds additional requirements. The first and foremost is fabrication of the microfluidic devices. Traditional microfluidics require the use of soft lithography molding of intricate fluid handling channels in clean rooms, followed by precise alignment of structures with expensive mask aligners (Xia and Whitesides, 1998). Large institutions may have access to such facilities, but not all laboratories do. Fortunately, most organ-on-a-chip devices do not require such high-resolution features, as they largely house and provide media flow to organoids or tissue constructs within. Therefore, lower resolution techniques can be used. This still includes soft lithography molding, but without the need for expensive equipment or other device fabrication methods such as stacking and folding of adhesive film layers or polymethylmethacrylate acrylic sheets with laser cut fluid channel and chamber features (Cooksey and Atencia, 2014; Aleman et al., 2019; Rajan et al., 2020a; Rajan et al., 2020b). Regardless of the fabrication technique, to operate organ-on-a-chip devices, there is often a need to connect tissue culture media reservoirs, tubing, and pumps, resulting in systems with many more moving parts and increased complexity compared to static organoid cultures that may simply be maintained in multi-well plates. Lastly, these microfluidic devices can occasionally have bubbles introduced, which can become lodged in regions of the devices, occluding flow and thus interrupting fresh tissue culture media availability to the cells, often resulting in decreased viability or complete experimental failure. In summary, as one moves from static organoid cultures to more dynamic organ-on-a-chip cultures, many new useful capabilities arise, but along with them come additional challenges to consider and manage.

## Final thoughts

Microphysiological systems are poised to make a significant contribution to how medicine and biomedical research are performed. In the United States, the FDA decision to remove the absolute requirement for animal model testing in the drug development pipeline serves as a loud signal that the world is open to new technologies and model systems to evaluate the toxicity and efficacy of therapeutics in development. Already, laboratories throughout the world have shown that, in organoids and organ-on-a-chip platforms, we can recapitulate toxicity outcomes observed caused by drug compounds that were removed from the market due to harming or killing human patients, as well as toxicity associated with environmental toxins (Forsythe et al., 2018; Skardal et al., 2020).

Personalized medicine is a clinical area where MPS can make a significant impact. For those of us actively working in this field, this is not a new concept, but for most, it is. We can readily see a clinical workflow where we utilize patient-derived cells to generate MPS specific to a patient. These patient-specific MPS will then be used to test multiple therapeutics to generate empirical data to inform clinicians on which therapeutic should be prescribed to achieve the most effective result with the least amount of side effects. We have already done this in select cases (Votanopoulos et al., 2019a), but there is much red tape to work through to make this standard practice at medical centers throughout the world.

Nevertheless, while organoid, organ-on-a-chip, and similar technologies have hurdles to overcome before universal acceptance and utilization, those of us working in-depth with these technologies stand poised to make a major impact in both biomedical research and clinical enterprises.
